# New insight into the dynamical system of *α*B-crystallin oligomers

**DOI:** 10.1038/srep29208

**Published:** 2016-07-06

**Authors:** Rintaro Inoue, Takumi Takata, Norihiko Fujii, Kentaro Ishii, Susumu Uchiyama, Nobuhiro Sato, Yojiro Oba, Kathleen Wood, Koichi Kato, Noriko Fujii, Masaaki Sugiyama

**Affiliations:** 1 Research Reactor Institute, Kyoto University, Kumatori-cho, Sennan-gun, Osaka 590-0494, Japan; 2 Teikyo Univ., Radioisotope Res. Ctr, Kaga Itabashi Ku, Tokyo, 173-8605, Japan; 3 Okazaki Institute for Integrative Bioscience, National Institutes of Natural Sciences, Okazaki 444-8787, Japan; 4 Graduate School of Engineering, Osaka University, Suita, Osaka, 565-0871, Japan; 5 Australian Nuclear Science and Technology Organization, Lucas Heights, NSW Australia

## Abstract

*α*-Crystallin possesses a dynamic quaternary structure mediated by its subunit dynamics. Elucidation of a mechanism of subunit dynamics in homo-oligomers of *α*B-crystallin was tackled through deuteration-assisted small-angle neutron scattering (DA-SANS) and electrospray ionization (ESI) native mass spectrometry (nMS). The existence of subunit exchange was confirmed with DA-SANS, and monomers liberated from the oligomers were observed with nMS. With increasing temperature, an increase in both the exchange rate and monomer population was observed despite the absence of oligomer collapse. It is proposed that transiently liberated subunits, namely, “traveling subunits,” play a role in subunit exchange. Moreover, we propose that protein function is regulated by these traveling subunits.

Most proteins are complexes composed of precisely positioned subunits. This subunit-assembled structure, known as the quaternary structure, statically and/or dynamically regulates the function of protein complexes. However, some protein oligomers, for example, small heat-shock proteins (sHSPs)^1^, have unique dynamical systems. In response to changes in the external environment, sHSPs undergo a drastic modulation of their quaternary structures, which includes the association/dissociation of constituting subunits. In other words, most oligomers that comprise sHSPs have neither fine nor fixed quaternary structures. Such a structural feature is considered to play a crucial role in protein function, although its underlying origin remains a matter of discussion.

*α*-Crystallin is an oligomer comprised primarily of 20–40 subunits of two types^2^, *α*A- and *α*B-crystallin, both with molecular masses of about 20 kDa. These subunits share consensus amino acid sequences with sHSPs. Because of the unavailability of its crystal structure, the determination of the quaternary structure of *α*-crystallin has been tackled with various state-of-the art experimental techniques^3^,^4^,^5^,^6^,^7^. However, no consensus on the revealed structures has been obtained. From this point of view, it has been suggested that *α*-crystallin has neither a fine nor a fixed quaternary structure. To unveil its enigmatic quaternary structure, a key concept could be “dynamic quaternary structure”. Proteins having this structure possess the following features. First, the association number and corresponding quaternary structure are averaged but dynamically fluctuating. Second, these proteins are strongly influenced by subtle changes in the environmental conditions. Third, these protein features are deeply connected to function. A plausible origin of dynamic quaternary structure may be derived from subunit dynamics between oligomers. Some experimental clues for subunit dynamics in *α*-crystallin have been reported by several groups^8^,^9^,^10^. Of these, van den Oetelaar *et al*.^8^ reported an intermolecular exchange of subunits in re-aggregated bovine *α*-crystallin, whereas Bova *et al*.^9^ studied subunit exchange in *α*A-crystallin oligomer through fluorescence resonance energy transfer. In addition, Baldwin *et al*. suggested that C-terminal region millisecond scale dynamics are responsible for subunit exchange in *α*B-crystallin oligomers through solution-state nuclear magnetic resonance spectroscopy (NMR) aided by NMR active spin labeling^10^. The clarification of the mechanism of subunit dynamics is necessary to reveal the correlation between the dynamic quaternary structure and the associated function in this biologically fascinating, significant system: the relationship between subunit dynamics and chaperone activity.

One of the most attractive properties in neutron scattering is its ability to discern hydrogen from deuterium through differences in neutron scattering lengths^11^. Deuterium labeling of biomolecules is more advantageous than other labeling techniques, such as site-directed mutagenesis, fluorescence, or chemical labeling, because of its low structural and dynamic perturbations. Deuteration-assisted small-angle neutron scattering (DA-SANS)^12^,^13^,^14^ presents a promising method to track subunit dynamics, such as subunit exchange between protein oligomers in solution. Furthermore, electrospray ionization (ESI) native mass spectrometry (nMS) is a suitable method to capture a snapshot of the precise distribution of the association number of protein oligomers^15^,^16^. Hence, the inherent features of subunit dynamics in protein oligomers can be pursued through the complementary use of DA-SANS and nMS. In this report, we investigate the subunit dynamics in homo-oligomers of *α*B-crystallin using the combination of DA-SANS and nMS and discuss its relevance to *α*B-crystallin’s biological functions.

## Results and Analyses

### Detection of subunit dynamics through DA-SANS

**Figure 1:**
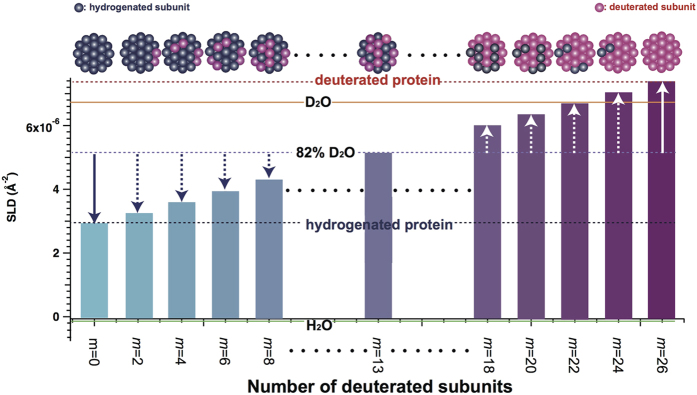
Schematic representation of scattering length density (SLD) as a function of the number of deuterated subunits *m* for a 26-mer of *α*B-crystallin. Red and blue circles correspond to deuterated and hydrogenated subunits, respectively. The orange solid, green solid, red dotted, blue dotted, and purple dotted lines correspond to SLD of D_2_O, H_2_O, d-*α*B, h-*α*B, and 82% D_2_O, respectively.

We addressed SANS intensity in solution mainly by focusing on forward scattering (*I*_0_). *I*_0_ is defined by the following equation.


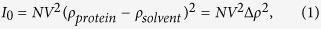


where *N*, *V*, *ρ*_protein_, and *ρ*_solvent_ correspond to the number density, volume, and scattering length densities (SLD) of protein and solvent, respectively, and Δ*ρ* (=*ρ*_protein_ − *ρ*_solvent_), is the difference in SLD between the protein and solvent. Based on previous works^3^,^4^,^5^,^6^,^7^, the average association number of *α*B-crystallin oligomer was estimated to be 26. In this work, the average association number of present *α*B-crystallin oligomer was also estimated to 26 from eq. (S1). (Supplementary Text). We then defined *ρ*_protein_ as a function of the number of average deuterated subunits *m* in Fig. 1. It should be noted that SLD of solvent is manipulated by changing the mixing ratio of H_2_O (*ρ*_solvent_ = −5.60 × 10^−7^ Å^−2^) and D_2_O (*ρ*_solvent_ = 6.37 × 10^−6^ Å^−2^) in neutron scattering. SLD of 82% D_2_O solvent (*ρ*_solvent_ = 5.11 × 10^−6^ Å^−2^) is an average of SLDs of hydrogenated 26-mers of *α*B-crystallin (h-*α*B, *m* = 0: *ρ*_protein_ = 2.86 × 10^−6^ Å^−2^) and deuterated subunits (d-*α*B, *m* = 26: *ρ*_protein_ = 7.35 × 10^−6^ Å^−2^). The contrasts for h-*α*B and d-*α*B in 82% D_2_O solvent are equal in absolute value but opposite in sign, as shown by the white and blue solid arrows in Fig. 1. In this report, 82% D_2_O solvent is an inverse contrast solvent, which was experimentally confirmed based on the fact that the scattering profile of h-*α*B coincided with that of d-*α*B (refer to Fig. S1). Assuming that subunit dynamics could occur in the mixture of h-*α*B and d-*α*B, *α*B-crystallin oligomers with different *m* values would be generated and the number distribution of *m* should be dispersed depending upon the exchange kinetics. Every *α*B-crystallin oligomer with *m* ≠ 0 or 26 has smaller |Δ*ρ*| than that of h-*α*B and d-*α*B in the inverse solvent, as shown by dotted blue and white arrows in Fig. 1. Hence, after mixing, *I*_0_ begins to decrease with the progress of subunit dynamics and ceases to decrease when the system reaches an isotopic equilibrium state. In other words, from the time evolution of *I*_0_, the progress of subunit dynamics in *α*B-crystallin oligomers can be tracked.

### Subunit exchange at 37 °C

**Figure 2:**
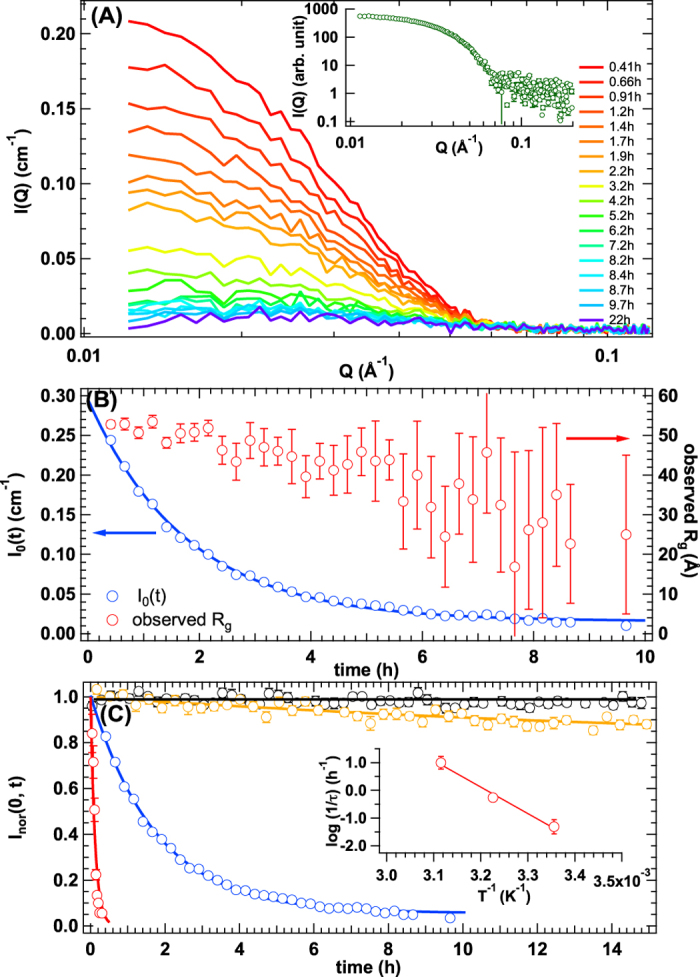
(**A**) Time evolution of small-angle neutron scattering profiles after mixing d-*α*B and h-*α*B in the inverse contrast solvent at 37 °C (red to purple lines correspond to 0.41 h to 22 h), and inset indicates small-angle X-ray scattering profile measured at 22 h after mixing d-*α*B and h-*α*B in the inverse contrast solvent at 37 °C. (**B**) Time dependence of *I*_0_ (blue circle) and observed *R*_g_ (red circle). The solid line corresponds to the result of fit with eq. (2). The direction and colour of arrows highlight the proper vertical axis for *I*_0_(*t*) and *R*_g_, respectively. (**C**) Time evolution of *I*_0_*_*_nor_(*t*) at 10 °C (black circle), 25 °C (yellow circle), 37 °C (blue circle), and 48 °C (red circle). The yellow, blue, and red curves correspond to the result of fit with eq. (2). Black line is guided by eye. Inset indicates Arrhenius plots of the relaxation rates.

Figure 2(A) shows the time evolution of SANS profile upon mixing h-*α*B and d-*α*B in the inverse contrast solvent at 37 °C. A clear decrease in *I*_0_ was observed with time. At this moment, there are two possibilities for the decrease in scattering intensity: the first is a decrease in the average contrast of *α*B-crystallin oligomers due to the subunit dynamics between them, which was expected, or second is their degradation upon mixing. To exclude the latter possibility, samples at 22 h after mixing, which showed almost no SANS intensity (purple line in Fig. 2(A)), were also measured by small-angle X-ray scattering (SAXS). The SAXS profile, as shown in the inset figure of Fig. 2(A), is similar to the SANS profiles from h-*α*B and d-*α*B (Fig. S1). *R*_g_ from SAXS profile was estimated to 54.5 ± 1.5 Å (Fig. S2(A)) and this value coincided with *R*_g_ of h-*α*B (=53.6 ± 1.1 Å) and d-*α*B (=53.2 ± 1.3 Å) within experimental error. In addition, the normalized Kratky plot from SAXS profile nicely coincided with that of h-*α*B and d-*α*B, as shown in Fig. S2(B). These results clearly support the absence of the degradation of *α*B-crystallin oligomer at 22 h after mixing. It is then concluded that the decrease in *I*_0_ in SANS measurements was attributed to subunit exchange between *α*B-crystallin oligomers. Next, we focused on the time evolution of *I*_0_, *I*_0_(*t*), because it reflects the kinetics of subunit exchange. As shown in Fig. 2(B), *I*_0_(*t*) can be described with a single exponential decay function, as follows:


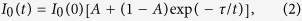


where *I*_0_(0), *τ*, and *A* correspond to *I*_0_ at *t* = 0, the decay time constant, and the ratio of decay to the initial intensity at the isotopic equilibrium state, respectively. This means that the subunit exchange apparently does not involve any intermediate states. Here, *τ* and *A* were calculated at 1.8 ± 0.043 h and 0.043 ± 0.0050, respectively. Based on our previous work^14^, *A* is related to the number of exchangeable subunits in an oligomer. It was found that almost all subunits in 26-mer *α*B-crystallin were exchanged (Supplementary Text and Fig. S3). We also considered the time evolution of the observed gyration radius (*R*_g_), which is sensitive to the spatial distribution of exchangeable subunits among the oligomers. As shown in Fig. 2(B), observed *R*_g_ was almost constant within 2 h of mixing, even though about 20% of the subunits of *α*B-crystallin oligomer had already been exchanged. This experimental finding indicates that the exchanged subunits were homogeneously distributed in the oligomer; that is, there is no spatial inhomogeneity in terms of subunit exchange.

### Temperature dependence of subunit exchange and averaged oligomeric structure

It is expected that subunit exchange would be affected by temperature change. Therefore, subunit exchange among *α*B-crystallin oligomers was examined at other three temperatures (10, 25, and 48 °C). The time evolution of SANS spectra is shown in Fig. S3. Figure 2(C) shows *I*_0_*_*_nor_(*t*)s normalized *I*_0_(*t*) by *I*_0_(0) at 10, 25, 37, and 48 °C, respectively. At 10 °C, no detectable decrease in *I*_0_*_*_nor_(*t*) was observed, indicating an extremely slow subunit exchange rate. With elevated temperature, an increase in the exchange rate was observed. Remarkably, all subunits were exchanged within 20 min at 48 °C. *I*_nor_(0, *t*)s at 25 and 48 °C were also well fitted with eq. (2), as shown in Fig. 2(C). From the temperature dependence of *τ*, the activation energy of subunit exchange was estimated at 19 ± 1.9 kcal/mol (inset of Fig. 2(C)). The evaluated activation energy is comparable to that of subunit dissociation energy^17^ or inter-exchange of phospholipid bilayer^12^. In addition, the average exchangeable subunit number in one *α*B-crystallin oligomer was estimated to be 0, 3, and all subunits at 10, 25, and 48 °C, respectively. The temperature dependence of the averaged oligomeric structure from the perspective of subunit exchange was also interesting. Figure S4(A) shows the temperature dependence of *R*_g_ of *α*B-crystallin oligomer. In a temperature range of 10 °C to 37 °C, *R*_g_ and the average association number remained constant, even though the exchange rates were different. When the temperature reached 48 °C, the situation was very different as the exchange rate increased drastically and the *R*_g_ value was also greater than that at the other temperatures. Temperature dependence of average association number of *α*B-crystallin oligomer is also plotted in Fig. S4(B). In parallel with the increase of *R*_g_, the average association number at 48 °C was greater than that at the other temperatures. We also examined the structure of the oligomers by dynamic light scattering (DLS) at 48 °C, as shown in Fig. S4(C). A clear mono-disperse distribution was confirmed from the result of CONTIN analysis, implying the absence of both abnormal aggregation and degradation. Then, the temperature dependence of *R*_h_ is also plotted in Fig. S4(D). Summarizing the temperature dependences of *R*_g_, *R*_h_ and average association number, it is suggested that the reorganization of the oligomeric structure observed at 48 °C is mainly attributed to increase of its association number.

### Snapshot of subunit exchange

**Figure 3:**
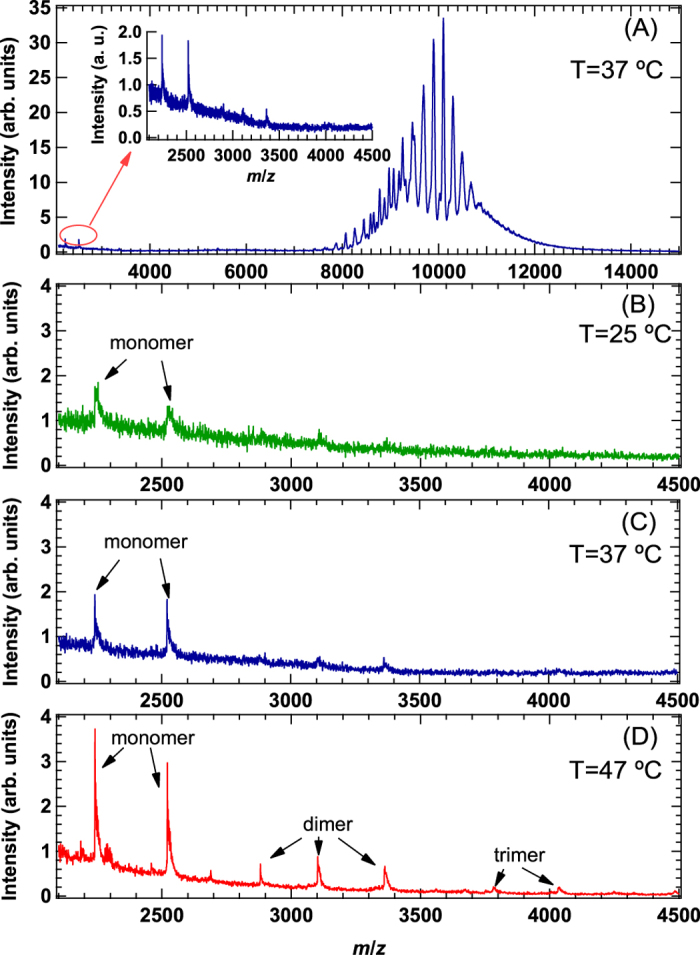
(**A**) Mass spectrum of *α*B-crystallin oligomer at 37 °C, and inset indicates the magnified spectrum at low *m*/*z* region ranging from 2100 to 4500. (**B**) Mass spectrum measured at 25 °C at low *m*/*z* region ranging from 2100 to 4500. (**C**) Mass spectrum measured at 37 °C at low *m*/*z* region ranging from 2100 to 4500. (**D**) Mass spectrum measured at 47 °C at low *m*/*z* region ranging from 2100 to 4500.

To understand the distribution of association number in *α*B-crystallin oligomers, nMS measurements were also performed at 25, 37, and 47 °C, respectively. As shown in Fig. 3(A), *α*B-crystallin oligomers were clearly observed between 7000 and 15000 *m*/*z* at 37 °C. At 25 and 37 °C similar distribution of association number in *α*B-crystallin oligomers were observed, on the other hand broad distribution of association number and increase of average association number was observed at 47 °C, as shown in Fig. S6. This trend is consistent with the results summarized in Fig. S5. Interestingly, a close inspection of the spectrum on the low *m*/*z* region, ranging from 2100 to 4500, revealed the existence of monomers (inset of Fig. 3(A) and Supplementary text). In addition, as shown in Fig. 3(B–D), the contribution of monomers increased with temperature, and even dimers and trimers were observed at 47 °C. However, no oligomers other than monomers, dimers, and trimers were detected, implying the absence of the collapse of *α*B-crystallin oligomers.

## Discussion

We initially assumed that subunit exchange in *α*B-crystallin oligomer proceeds through the collision between two *α*B-crystallin oligomers (bimolecular collision exchange model) (supplementary text and Figs S7–S9). This model does not support the experimental results adequately; hence we have to consider other possibility. Then, we summarize the experimental results obtained from DA-SANS and nMS to propose a plausible mechanism of subunit exchange among *α*B-crystallin oligomers.

**Figure 4:**
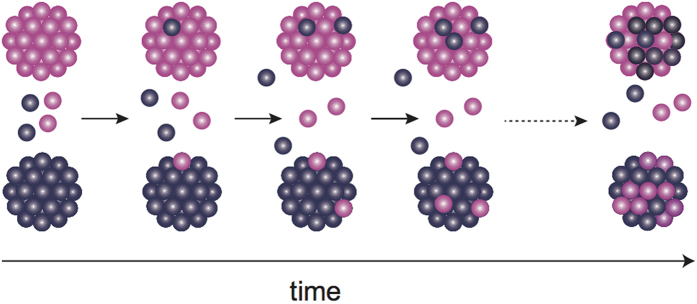
Schematic representation of subunit exchange among *α*B-crystallin oligomers. The large grape-like clusters and small grape-like spheres correspond to *α*B-crystallin oligomer and traveling subunits, respectively.

First, only monomers, in addition to normal-sized oligomers, existed in solution under physiological conditions and the oligomer subunits were exchanged. Second, the monomer population increased with the increase in temperature and exchange rate. Third, the average size (*R*_g_) and number of oligomers increased, whereas the exchange rate accelerated drastically. Moreover, monomer contribution was also observed at 48 °C with an enhanced chaperone activity^18^. Taking into account the first and second results, the liberated monomers (or subunits) from one oligomer are transferred to another in subunit exchange. In other words, such liberated monomers serve as “traveling subunits.” With increasing temperature, the number of subunits exceeding the activation energy is expected to increase. Then, the encountering probability between the traveling subunits and oligomers will increase with temperature, leading to an increased exchange rate with temperature; thus, the traveling subunits must be the main contributors to subunit exchange among *α*B-crystallin oligomers (Fig. 4).

It is considered that dissociated small oligomers are a key factor for the regulation of sHSP chaperone activity^1^. In fact, it was reported that their dissociation into smaller oligomers is necessary to activate chaperone activity for wheat HSP 16.9^19^. As discussed in the previous section, it was confirmed that traveling subunits exist in *α*B-crystallin oligomer solution, and therefore, it is considered that they play a leading role in chaperone activity as follows: (1) traveling subunits can effectively expose hydrophobic surfaces to allow preferred hydrophobic interactions with unfolded/partially unfolded target proteins; and (2) the increased contribution of traveling subunits can enhance the probability of capturing the target protein. Interestingly, the existence of *α*-crystallin has been reported at the downstream of the transcription initiation site on the *γ*-crystallin^20^ though the molecular cut-off of nuclear membrane was at around 70 kDa^21^. Recently, some research groups^22^,^23^ reported that the monomeric *α-*crytsallin domain possesses the proper chaperone activity. In addition, Horwitz *et al*.^24^ reported the absence of difference in the chaperone activity between an oligomer and a monomer of *α*B-crystallin. These insightful results support that the chaperone activity of *α*B-crystallin is regulated by monomers, supporting the concept of activation of chaperone activity by traveling subunits. On the other hand, Doss *et al*.^25^ discussed that the exposing hydrophobic surface is not favourable from the point of view of solubility. It is then expected that the liberated monomers will return to the oligomers after traveling and induce “subunit exchange.” In other words, the coexistence of traveling subunits and normal oligomers in *α*B-crystallin are biologically required for balancing chaperone activity and proper solubility.

At temperatures greater than 46 °C, increased exposure of solvent-exposed hydrophobic surfaces was reported for *α*-crystallin^26^. Under such conditions, it is assumed that the hydrophobic interactions between *α*-crystallins would be strengthened along with the interaction between *α*-crystallin and target protein, thereby contributing to the increase in the association number and size of *α*-crystallin. This assumption is consistent with the third summarized result in the previous section. It is considered that both the increased exposure of hydrophobic surfaces and number of traveling subunits are responsible for chaperone activity at high temperatures.

## Materials and Methods

### Overexpression and purification of human recombinant hydrogenated/deuterated *α*B-crystallin oligomer

Recombinant human *α*B-crystallin was subcloned into *Nco*I/*Hin*dIII sites of the vector pET-23d(+) (Novagen-Merck Biosciences, Ltd., Nottingham, UK). *α*B-crystallin plasmids were transformed into *E. coli* strain BL21 (DE3)pLysS cells and expressed as recombinant *α*B-crystallin. For the preparation of hydrogenated *α*B-crystallin oligomers, *E. coli* transformants were cultured in 5-mL Luria-Bertani (LB) culture solution containing 50-*μ*g/mL ampicillin for 12 h at 37 °C. Next, 1.0 mL of this solution was added to 1 L of fresh LB medium and further cultured for approximately 4.5 h (OD_600_ = 0.6) at 37 °C, after which *α*B-crystallin expression was induced by the addition of isopropyl-1-thio-*β*-D-galactopyranoside (IPTG) at a final concentration of 0.3 mM, and the cells grown for an additional 5 h at 25 °C. For the preparation of deuterated *α*B-crystallin oligomer, *E. coli* transformants were first cultured in 5-mL LB culture solution dissolved in 30% D_2_O containing 50-mg/mL ampicillin for 12 h at 37 °C. Further, 50 *μ*L of this solution was added to 5-mL LB medium dissolved in 60% D_2_O containing 50-*μ*g/mL ampicillin and cultured for 12 h. Subsequently, 10 *μ*L of this LB medium was added to 10 mL of LB culture medium dissolved in 80% D_2_O containing 50-*μ*g/mL ampicillin and cultured for 12 h. The cells were then collected by centrifugation at 7000 rpm for 15 min at 4 °C and resuspended in M9 minimal media containing deuterated glucose (2 g/L) and 99.8% D_2_O. The cells were cultured for approximately 28 h (OD_600_ = 0.6) at 37 °C, after which the expression of *α*B-crystallin was induced by the addition of IPTG at a final concentration of 0.3 mM, and the cells grown for an additional 10 h at 25 °C. *E*. *coli* cells grown in both LB and minimal M9 media were sonicated in 20 mM Tris-HCl (pH 7.8), 1.0 mM ethylenediaminetetraacetic acid (EDTA), and 1.0 mM phenylmethanesulfonyl fluoride. The cell lysates were then centrifuged at 12000 rpm for 20 min at 4 °C to separate the supernatant from the cellular pellet. The supernatant was applied to an ion-exchange column (Q Sepharose XL, Amersham BioSciences, Little Chalfont, UK) equilibrated in 20 mM Tris-HCl (pH 7.8) and 1.0 mM EDTA and eluted with a linear gradient of 0–1 M NaCl at a flow rate of 10.0 mL/min. *α*B-crystallin oligomers were fractioned, concentrated, and filtered through a 0.45-*μ*m membrane before applying to a gel filtration column (HiPrep 16/60 Sephacryl S-300 HR; GE Healthcare, Little Chalfont, UK) equilibrated with 20 mM Tris/HCl buffer (pH 7.8) and 150 mM NaCl. The purity of *α*B-crystallin oligomers was confirmed by sodium dodecyl sulfate polyacrylamide gel electrophoresis and concentrated using an Amicon spin concentrator (EMD Millipore, Billerica, MA, USA). The concentrated *α*B-crystallin oligomers were dialyzed against 20 mM Tris/HCl (pH 7.4/pD 7.0), 150 mM NaCl, and H_2_O/D_2_O buffer and filtered with an Ultrafree-MC VV Centrifugal Filter (Merck Millipore Ltd., Cork, Ireland) before use. The final concentration of hydrogenated and deuterated *α*B-crystallin oligomers after the purification procedure was estimated to be 0.89 mg/mL from the UV absorbance at 280 nm. The extinction coefficient for *α*B-crystallin was found to be 0.693 (mg/mL)^−1^cm^−1^.

### Small-angle neutron scattering (SANS)

SANS experiments were performed with Quokka installed at the Australian Nuclear Science and Technology Organization (ANSTO, Lucas Heights, NSW, Australia), Bio-SANS installed at the Oak Ridge National Laboratory (Oak Ridge, TN, USA), and TAIKAN installed at the Japan Proton Accelerator Complex (Tokai, Japan). For time-resolved SANS measurements, SANS intensities were measured at 15-min intervals at 10 and 37 °C. The acquisition time was 20 min and 2 min at 25 °C and 48 °C, respectively. The obtained SANS intensity was corrected for background, empty cell, buffer solution scattering, and transmittance. The SANS profiles thus obtained were converted to absolute intensities (cm^−1^) using standard samples or the direct beam method.

### Small-angle x-ray scattering (SAXS)

The SAXS experiments were performed using the NANOSTAR, Bruker installed at ANSTO. The obtained SAXS intensity was corrected for background, empty cell, buffer solution scattering, and transmittance.

### Dynamic light scattering (DLS)

DLS measurements were obtained using a system equipped with a 22-mW He-Ne laser, an Avalanche Photo Diode (APD, ALV, Germany) mounted on static/dynamic compact goniometer, ALV/LSE-5003 electronics, and ALV-5000 Correlator (ALV-Laser Vertriebsgesellschaft GmbH, Langen, Germany). The measurements were obtained at 10, 25, 37, and 48 °C. CONTIN analysis was used to obtain the probability of decay rate.

### Electrospray ionization native mass spectrometry (nMS)

The samples (1 mg/mL) were incubated at target temperature (25, 37, and 47 °C) for 30 min and then were buffer-exchanged in 150 mM ammonium acetate (pH 7.5) by passing the proteins through a Bio-Spin 6 column (Bio-Rad Laboratories, Hercules, CA, USA). The buffer-exchanged samples were immediately analyzed by nanoflow electrospray ionization mass spectrometry using gold-coated glass capillaries made in-house (approximately 2-μL sample was loaded per analysis). Spectra were recorded on a SYNAPT G2-S*i* HDMS mass spectrometer (Waters, Manchester, UK) in positive ionization mode at 1.33 kV with a 150-V sampling cone voltage and source offset voltage, regulated cone temperature, 0-V trap and transfer collision energy, and 5-mL/min trap gas flow. The spectra were calibrated using 1-mg/mL cesium iodide and analyzed using Mass Lynx software (Waters).

## Additional Information

**How to cite this article**: Inoue, R. *et al*. New insight into the dynamical system of *α*B-crystallin oligomers. *Sci. Rep.* **6**, 29208; doi: 10.1038/srep29208 (2016).

## Supplementary Material

Supplementary Information
