# Supplementary material for: New insight into the dynamical system of αB-crystallin oligomers
Source: Sci Rep. 2016 Jul 6;6:29208. doi: 10.1038/srep29208 (PMC4933968; doi:10.1038/srep29208)
Supplement: Supplementary Information [file srep29208-s1.pdf]

Supplementary Materials

New insight into the dynamical system of αB-crystallin oligomers

Rintaro Inoue*, Takumi Takata, Norihiko Fujii, Kentaro Ishii, Susumu Uchiyama, Nobuhiro Sato, Yojiro Oba, Kathleen Wood, Koichi Kato, Noriko Fujii & Masaaki Sugiyama*

correspondence to: R. I. (rintaro@rri.kyoto-u.ac.jp), M. S. (sugiyama@rri.kyoto-u.ac.jp)

This PDF file includes:

Supplementary Text

Figs. S1 to S9

Supplementary Text

Small-angle neutron scattering profiles of hydrogenated and deutearted αB-crystallin oligomers at 37 °C

The scattering profile of hydrogenated αB-crystallin oligomers (h-αB) coincided with that of deuterated αB-crystallin oligomers (d-αB) in the inverse contrast solvent at 37°C, as shown in Fig. S1. The radii of gyration (R_g) and $I(0)$ of h-αB and d-αB were evaluated from Guinier analysis. The evaluated R_g values of h-αB and d-αB were 53.6 ± 1.1 Å and 53.2 ± 1.3 Å, respectively. Here, N in eq. (1) is given by the following equation:

$$N = cN_A/M_w, \tag{S1}$$

where c, N_A, and M_w are the concentration of protein in weight %, Avogadro's number, and molecular weight, respectively. In addition, the volume of αB-crystallin in solution can be calculated based on the amino acid sequence[1]. Then, M_w, which is comparable to the aggregation number of h-αB and d-αB, can be arithmetically calculated from I_0. The average number of subunits in h-αB and d-αB was calculated to be 26. This result supports that the expected contrasts from h-αB and d-αB were experimentally fulfilled and no structural difference existed between them.

Evaluation of the number of exchangeable subunits in αB-crystallin oligomer at 37 °C

We assumed that all subunits in the 26-mer αB-crystallin had an equal probability of exchanging randomly with any other subunit in the other oligomer. Isotopically heterogeneous oligomers comprising hydrogenated and deuterated subunits would be generated with the progress of time. Under the constraint of the number of exchangeable subunits n, we calculated the number of deuterated subunits x in isotopically mixed 26-mers of αB-crystallin and A_n in the equilibrium states as follows:

$$A_n = \sum_{x=0}^{n} \frac{{}_nC_x}{2^n}\left(\frac{26-2x}{26}\right)^2$$

(S2)

Considering the error bar of experimentally obtained A, almost all subunits of the 26-mer αB-crystallin were found to exchange at 37°C (Please refer to Fig. S3).

Detection of αB-crystallin monomer from nMS spectra.

Electrospray ionization (ESI) native mass spectrometry was used for the analysis of oligomeric state of αB-crystallin. The ESI tends to enable proteins highly charged states, which provides multi-valence peaks in mass spectrum even for one protein. In Fig. 3, the peaks of *m/z* 2240 and 2520 are corresponded to +9 and +8 charged states of αB-crystallin, respectively. From these ion series, the mass was estimated to 20159 Da, which is well consistent with the calculated molecular mass (20146 Da) of αB-crystallin monomer.

Bimolecular collision exchange model.

Firstly, we assumed that the subunit exchange could be described by random collision between two αB-crystallin oligomers (bimolecular collision exchange model), as shown in Fig. S7. Under such an assumption, it is considered that subunit exchange as a function of collision event step (m) can be given by the following equations.

$$I_{0\_nor}(m) = \sum_{N=0}^{r} f_N(m)\frac{(N\Delta\rho-(r-N)\Delta\rho)^2}{r^2\Delta\rho^2} = \sum_{N=0}^{r} f_N(m)\frac{(r-2N)^2}{r^2}$$

(S3)

$$\sum_{N=0}^{r} f_N(m) = 1$$

(S4)

where $r, m, N, \Delta\rho, f_N$ correspond to average association number (26 or 40), collision event step, number of deuterated subunits in αB-crystallin oligomer, scattering contrast and fraction of deuterated subunit (N) in

αB-crystallin oligomer. In order to reproduce experimentally obtained time dependence of $I_{0\_nor}(t)$, the appropriate collision frequency (k) was selected. The dotted curves in Fig. S8 indicate the best fitted ones for the description of time dependence of $I_{0\_nor}(t)$ at 25, 37 and 48 ºC. It should be noted that the collision frequency could be evaluated from experimentally obtained parameters such as diffusion coefficient (D), sample concentration and so on, assuming the diffusion-limited reaction[2]. Fig. S9 shows the temperature dependence of collision frequency from above-simulation and experiment technique (C_dc). It can be clearly seen that temperature dependence of collision frequency (C_dc) differ from k, suggesting that subunit exchange of αB-crystallin cannot be explained under bi-molecular collision process.

Reference

1. Jacrot, B. & Zaccai, G. Determination of molecular weight by neutron scattwring. *Biopolymers,* **20**, 2413-2426 (1981).
2. Smoluchowski, M. V. Versuch einer mathematischen theorie der koagulationskinetik kolloider lösungen. *Z. Physik. Chem.,* **92**, 129 (1917).

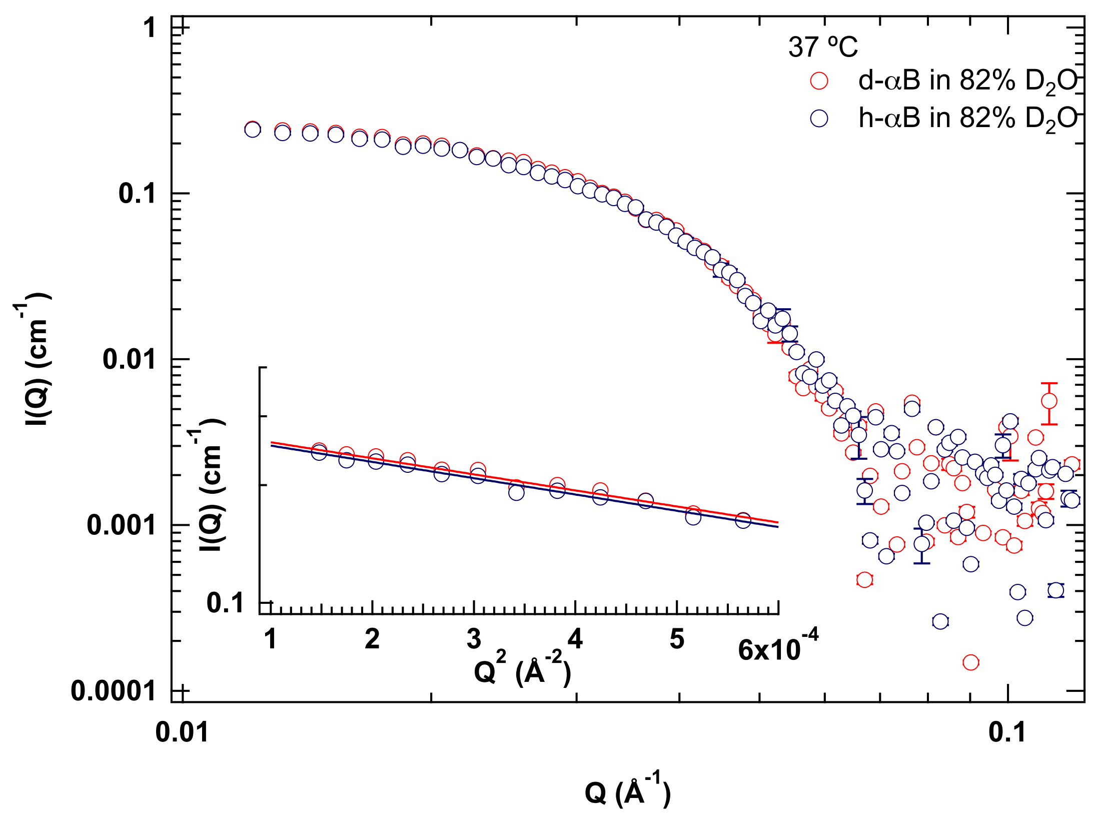

Fig. S1. Small-angle neutron scattering profiles from d-αB (red circle) and h-αB (blue circle) in the inverse contrast solvent at 37°C. Inset indicates Guinier plots of d-αB and h-αB, and the solid lines correspond to the results of the least-squared fit with Guinier function.

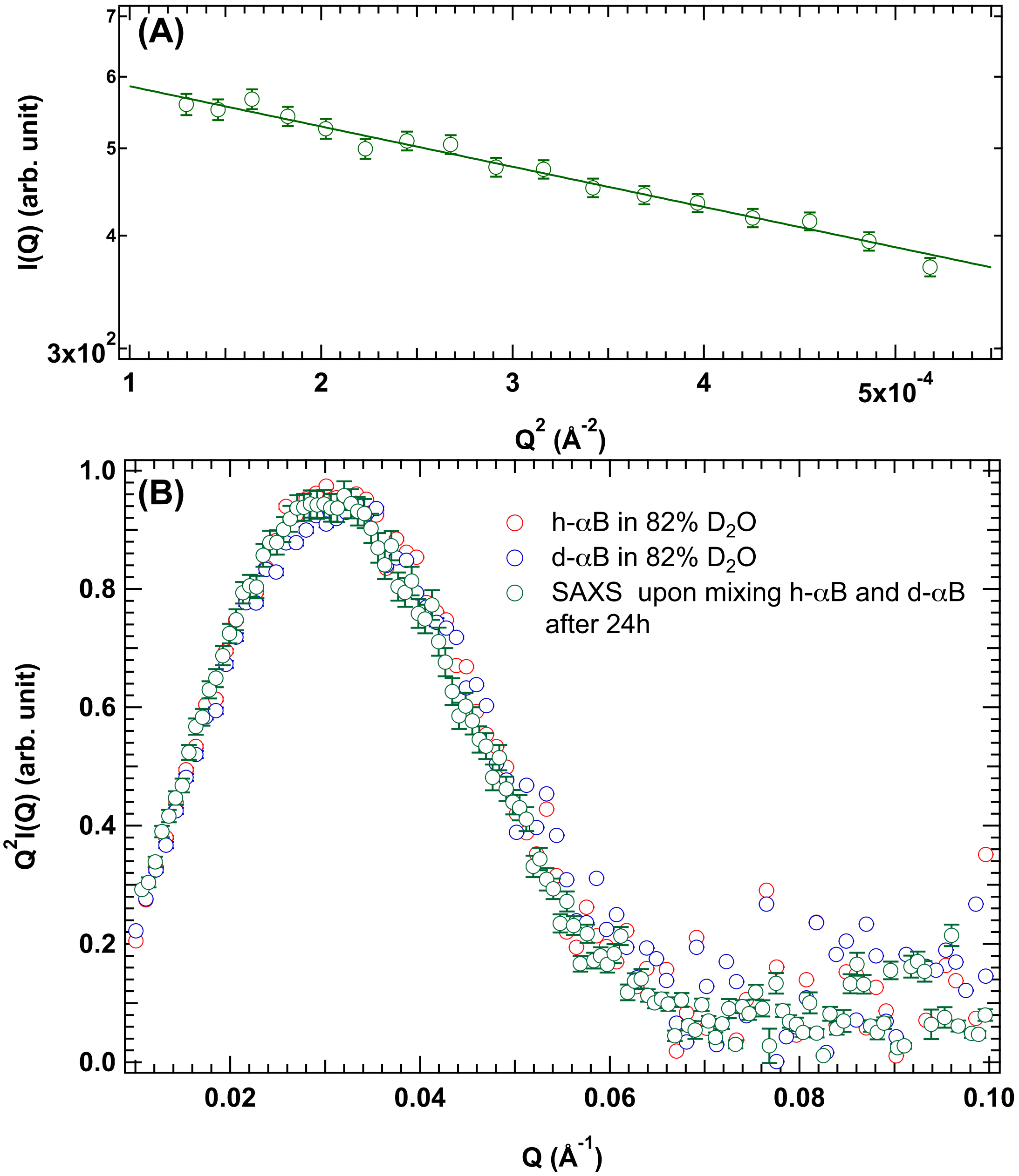

Fig. S2. (A) Guinier plot of SAXS profile at 22h after mixing d-αB and h-αB in the inverse contrast solvent at 37°C. (B) Normalized Kratky plot of SAXS (green circle), d-αB (blue circle) and h-αB (red circle).

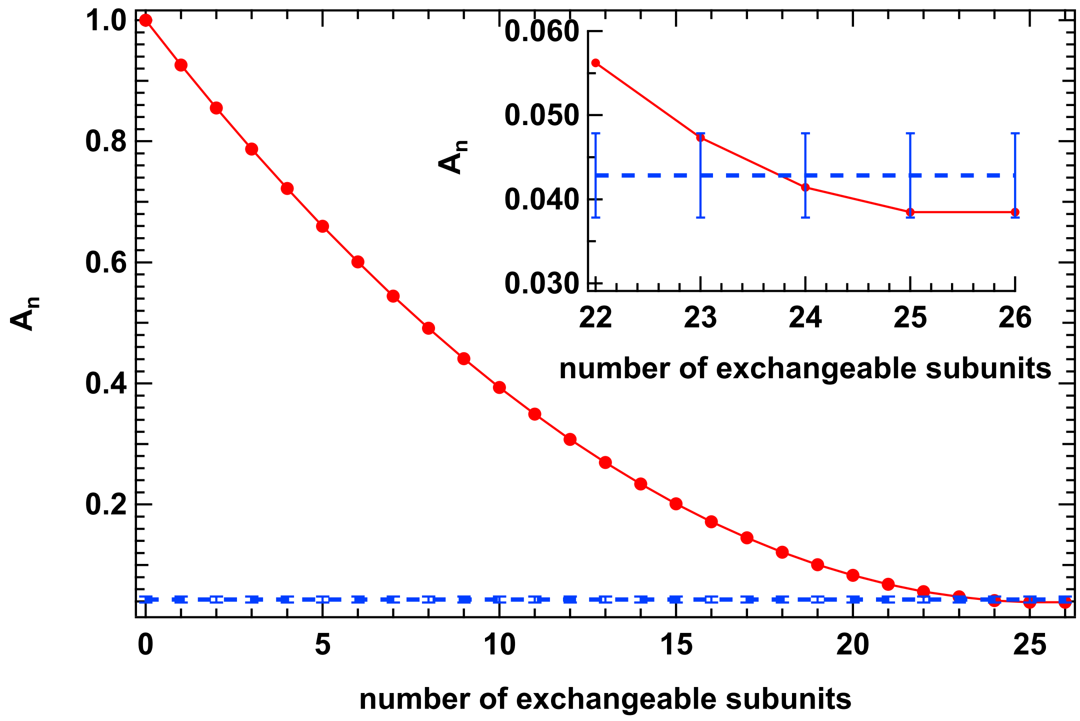

Fig. S3. A_n as a function of the number of exchangeable subunits for 26-mer of αB-crystallin. The blue dotted line corresponds to A obtained from eq. (2) at 37°C. Inset indicates the magnified figure.

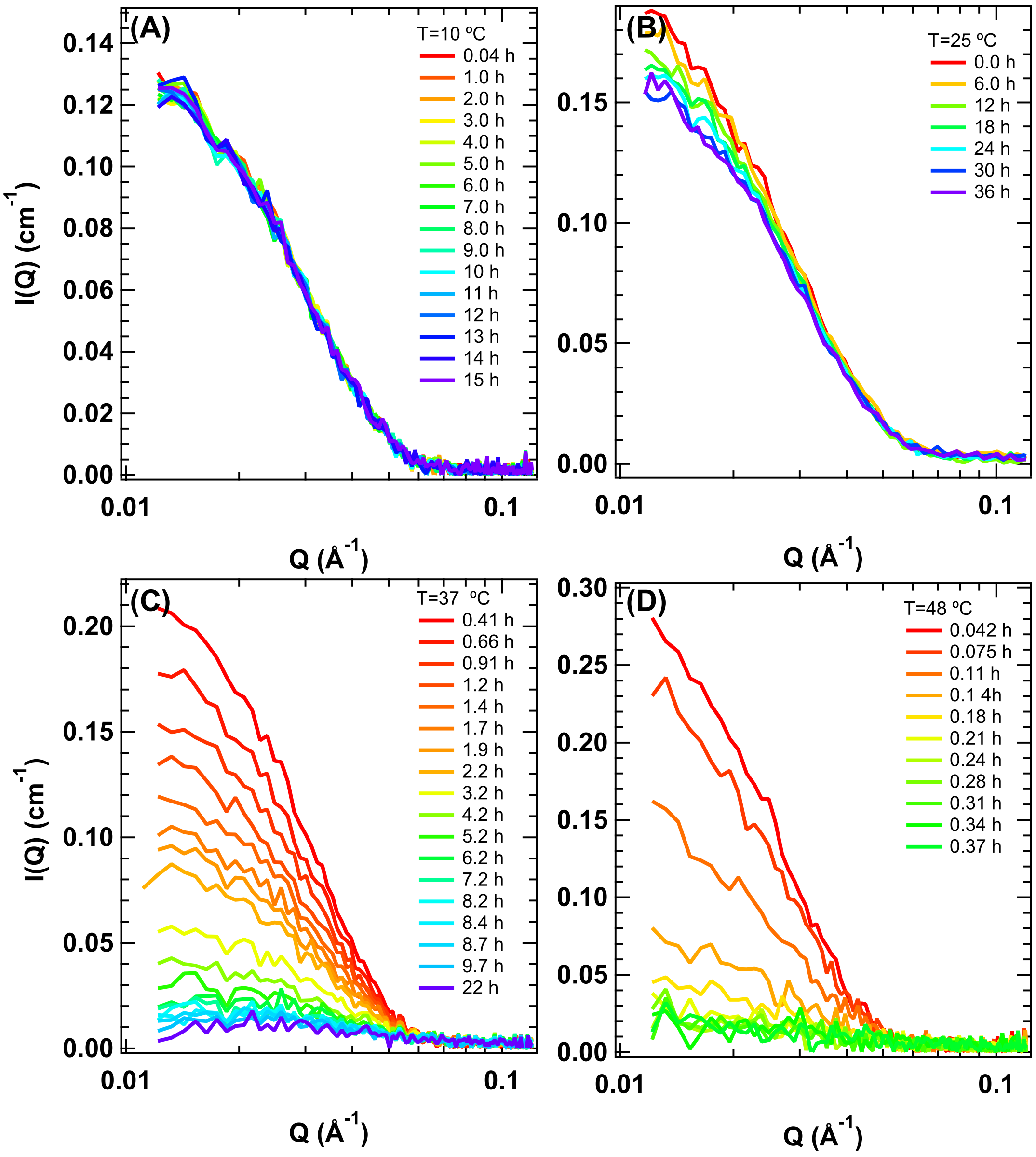

Fig. S4. Time evolution of small-angle neutron scattering profiles upon mixing d-αB and h-αB in the inverse contrast solvent at (A) 10°C (red to purple lines correspond to 0.040 to 15 h), (B) 25°C (red to purple lines correspond to 0.00 to 36 h), (C) 37°C (red to purple lines correspond to 0.41 to 22 h), and (D) 48°C (red to green lines correspond to 0.042 to 0.37 h).

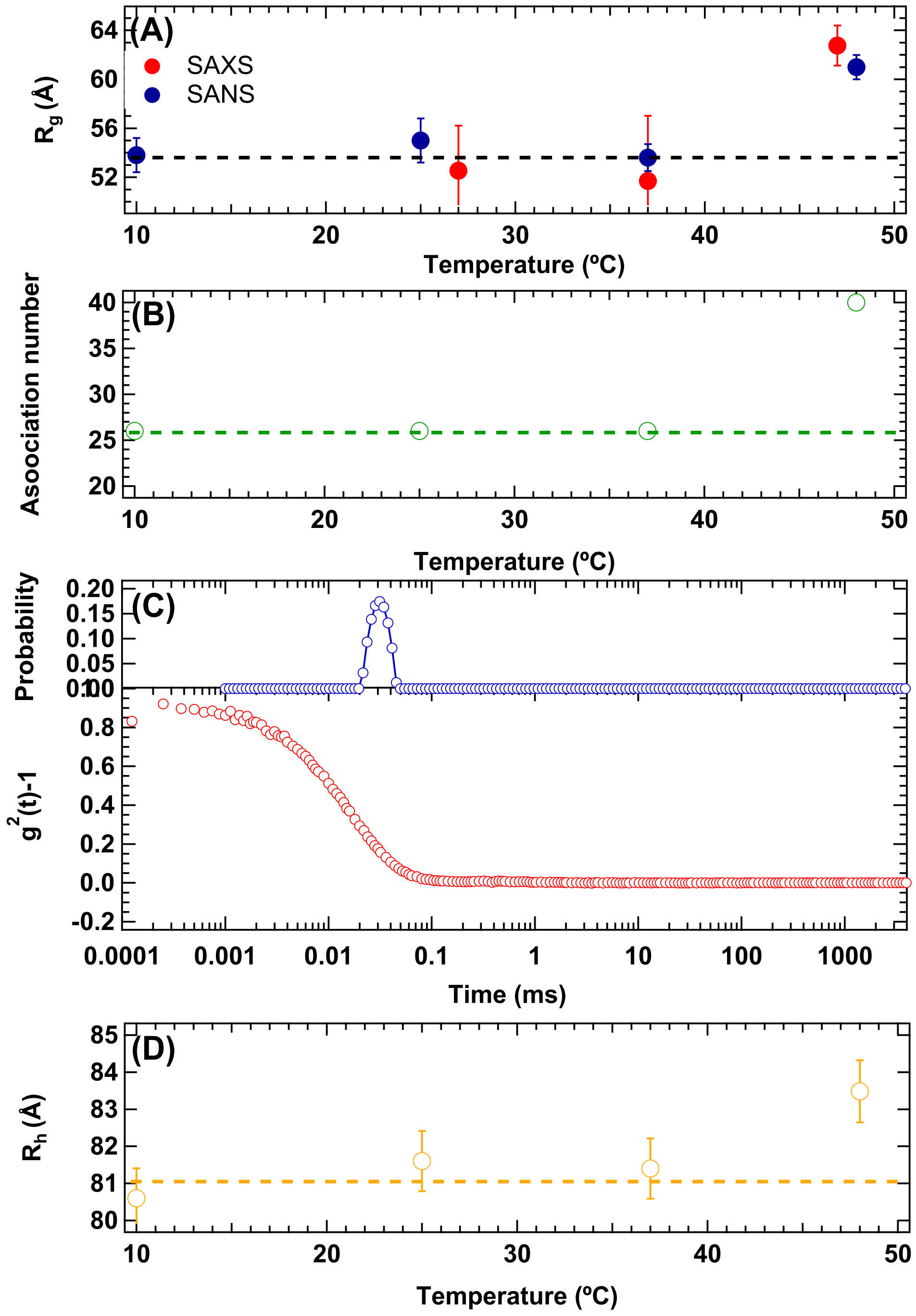

Fig. S5. (A) Temperature dependence of R_g evaluated from small-angle neutron scattering (blue circle) and small-angle X-ray scattering (red circle) measurements. (B) Temperature dependence of association number of oligomeric αB-crystallin. (C) The upper panel corresponds to the CONTIN analysis results of the intensity correlation function $g^2(t)-1$ at $Q = 2.56 \times 10^{-3}$ Å^{-1} at 48°C. The lower panel corresponds to $g^2(t)-1$ at $Q = 2.56 \times 10^{-3}$ Å^{-1} at 48°C. (D) Temperature dependence of R_h evaluated from DLS.

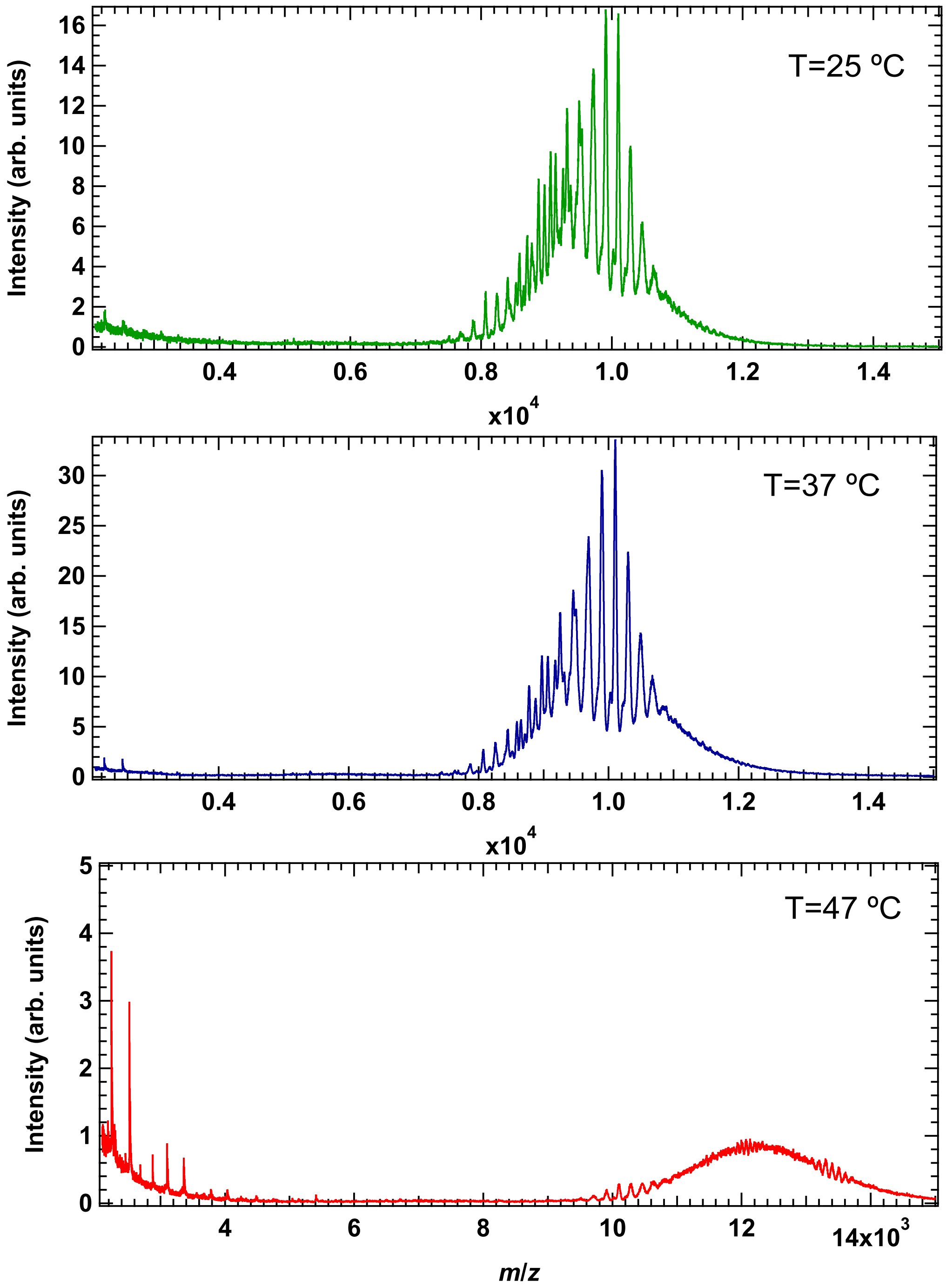

Fig. S6. (A) Mass spectrum of αB-crystallin oligomer at 25°C. (B) Mass spectrum of αB-crystallin oligomer at 37°C. (C) Mass spectrum of αB-crystallin oligomer at 47°C.

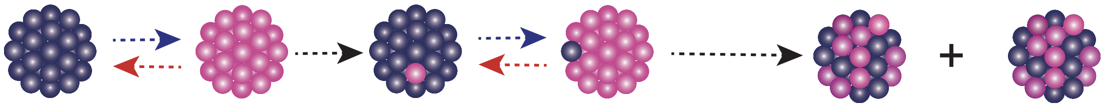

Fig. S7. Schematic view of bi-molecular collision exchange model.

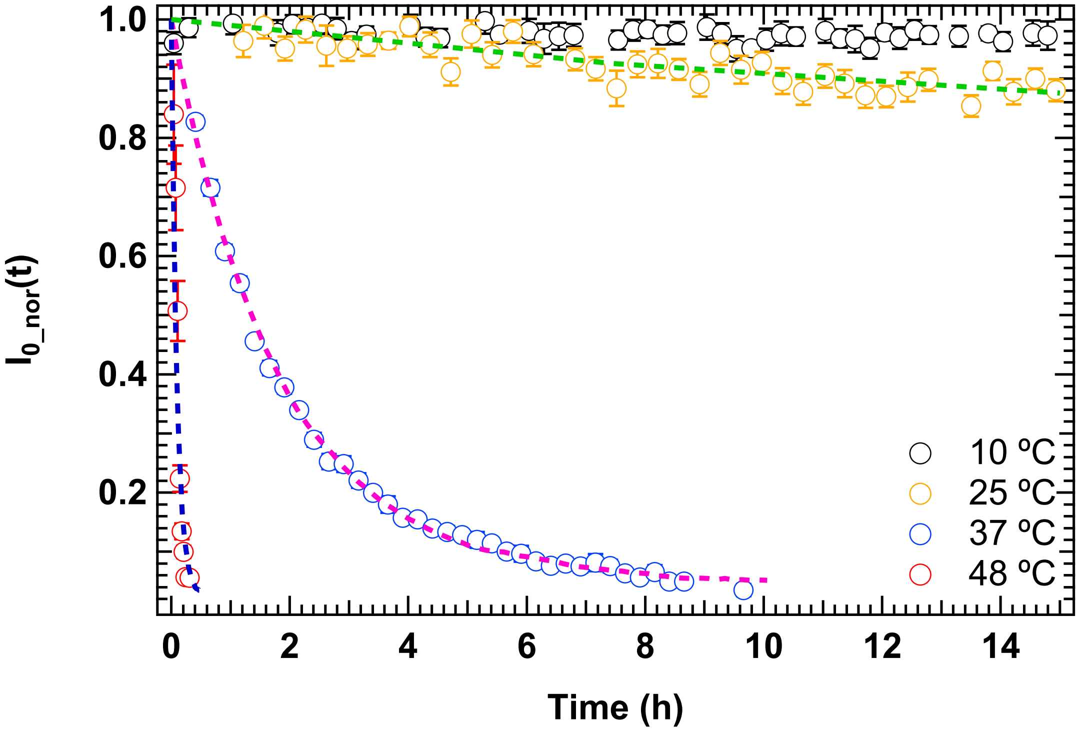

Fig. S8. Time evolution of $I_{0\_nor}(t)$ at 10°C (black circle), 25°C (yellow circle), 37°C (blue circle), and 48°C (red circle). The green, pink, and blue dotted curves correspond to the best-simulated curves based on bi-molecular collision exchange model.

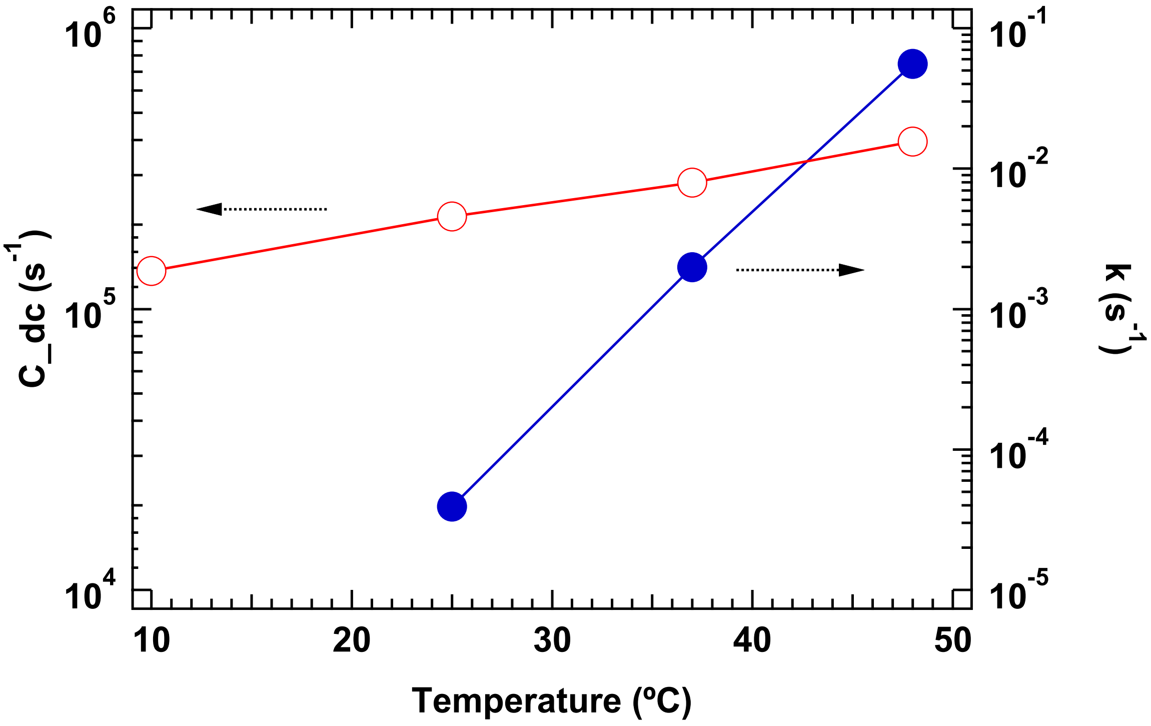

Fig. S9. Temperature dependence of collision frequency (*k*) (blue filled circle) calculated from bi-molecular collision exchange model and collision frequency (C_dc) (red circle) assuming diffusion limited reaction. The direction of dotted arrows correspond to the proper vertical axis.
